# Synergistic Li/Li Bimetallic System for the Asymmetric Synthesis of Antituberculosis Drug TBAJ-587

**DOI:** 10.1021/acs.joc.3c00705

**Published:** 2023-05-01

**Authors:** Tanveer Ahmad, Feng Gao, Jing Li, Zhenfeng Zhang, Tao Song, Qianjia Yuan, Wanbin Zhang

**Affiliations:** † Frontiers Science Center for Transformative Molecules, School of Chemistry and Chemical Engineering, Shanghai Jiao Tong University, 800 Dongchuan Road, Shanghai 200240, China; ‡ Shanghai Key Laboratory for Molecular Engineering of Chiral Drugs, School of Pharmacy, Shanghai Jiao Tong University, 800 Dongchuan Road, Shanghai 200240, China

## Abstract

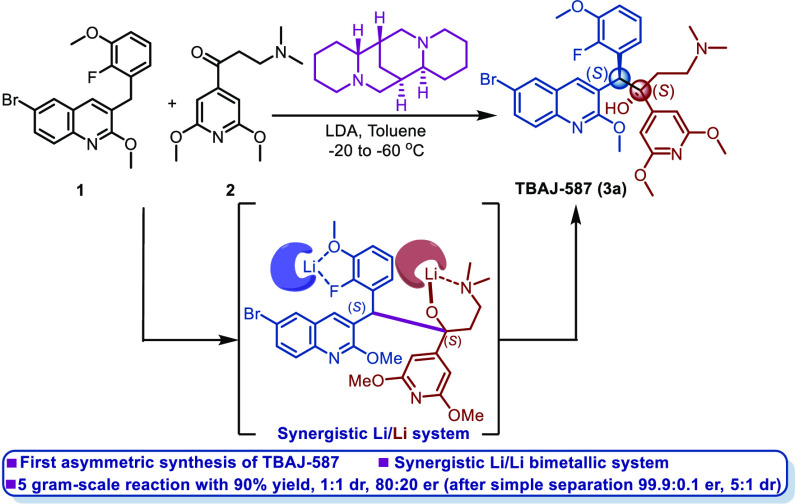

TBAJ-587, an analogue of the antituberculosis drug bedaquiline (BDQ), bearing a diarylquinoline skeleton retains the high bacterial potency, is less toxic, and has a better pharmacokinetic profile than the parent molecule, which has entered phase I clinical trials. In contrast to its fascinating bioactivity, however, the highly efficient synthesis of this molecule is still an unsolved challenge. Herein, the first asymmetric synthesis of TBAJ-587 based on a synergistic Li/Li bimetallic system is reported. The product could be obtained in an excellent yield of 90% and an enantiomeric ratio (er) of 80:20. Furthermore, the reaction could be conducted on a 5 g scale, and the product was obtained with 99.9:0.1 er after a simple recrystallization. The realization of this protocol will greatly aid the demand for clinical drug production.

Tuberculosis (TB), still poses a global threat as one of the deadliest infectious diseases.^1^ Moreover, multidrug-resistant TB (MDR-TB) and extensively drug-resistant TB (XDR-TB) are spreading worldwide, causing major global health issues.^2^ Therefore, much effort has been made to develop effective new drugs for the treatment of TB.^3^ As a result, the first MDR antituberculosis drug bedaquiline (BDQ), a new ATP synthase inhibitor, was approved by the FDA in 2012 as the first antituberculosis-specific drug.^4^ This also paved the way for medicinal chemists to discover new ATP synthase inhibitors that were more effective and potent against MDR/XDR-TB.^5^ To our delight, TBAJ-587, a new generation of BDQ derivatives developed by TB Alliance and currently undergoing clinical studies, was found to be as potent as BDQ against the pathogen *Mycobacterium tuberculosis*.^6^

The stereoselective formation of C(sp^3^)–C(sp^3^) bonds to generate vicinal stereocenters is important but challenging.^7^ In particular, the efficient construction of a chiral tertiary alcohol that contains contiguous tertiary and tetrasubstituted carbons is generally regarded as exceptionally difficult in organic synthesis.^8^ In addition to the common challenges associated with the construction of tertiary alcohol stereocenters, the chiral tertiary alcohols themselves are susceptible to racemization.^8^ In particular, the molecule TBAJ-587 (Scheme 1a), a chiral tertiary alcohol, contains vicinal stereocenters and is prepared only through the addition of quinoline **1** to aryl ketone **2** under basic conditions. The nucleophilic addition reaction of aryl ketones represents a formidable challenge in synthetic chemistry due to the attenuated reactivity and steric hindrance.^8^ The only synthetic protocol of TBAJ-587 reported so far provides an isomeric mixture with a yield of only 52%, no enantioselectivity, and poor reproducibility (Scheme 1a).^9^ Thus, the development of general, efficient, and atom-economical strategies to furnish TBAJ-587 is highly desirable.

**Scheme 1:**
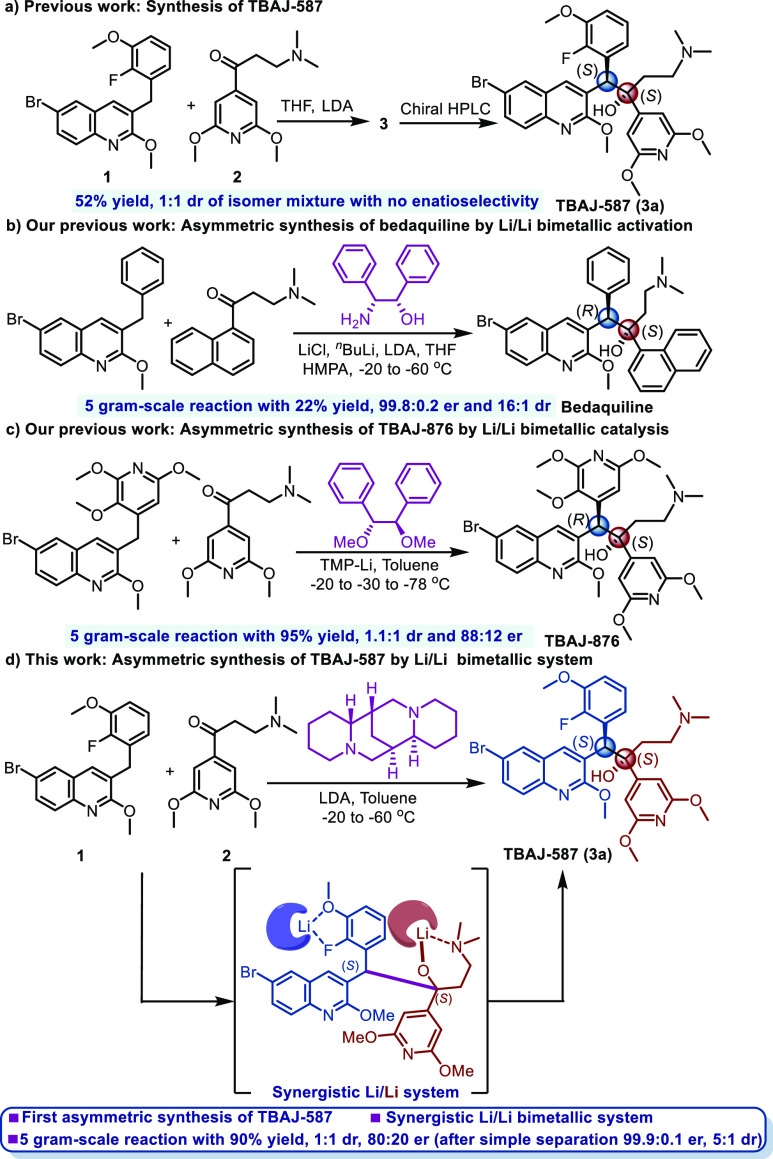
Synthesis of BDQ and Its Analogues

In the past few years, our group has started a research program studying synergistic bimetallic catalysis,^10,11^ which is one of the most powerful tools for constructing C(sp^3^)–C(sp^3^) bonds and has also been evidenced by many other elegant pieces of work.^12,13^ Very recently, we have developed a synergistic Li/Li bimetallic system to address the problems concerning the nucleophilic addition reaction of aryl ketones. The bimetallic system combined with noncovalent interactions has been shown to be a compelling strategy for the asymmetric synthesis of antituberculosis drugs BDQ and TBAJ-876 bearing contiguous chiral centers (Scheme 1b and 1c).^11^ Furthermore, we have established a library of known ligands for the synthesis of these types of compounds. However, the development of effective methods for the asymmetric synthesis of TBAJ-587 is even more difficult for the following reasons: (1) compared with BDQ, the corresponding starting material of TBAJ-587 contains additional coordinating groups (−F and −OMe) that coordinate readily with metal ions, resulting in a more complex coordination mode; (2) compared with TBAJ-876, it contains only one pyridine ring and so there are no more heteroatoms that can coordinate with the metal ion according to resonance theory. Consequently, the synthetic systems of both BDQ and TBAJ-876 are not suitable for the asymmetric synthesis of TBAJ-587.

In a continuation of our interest in exploring Li/Li catalytic systems and their application in the asymmetric synthesis of BDQ analogues, we envisioned that a suitable synergistic system and noncovalent interactions could address the above challenges regarding TBAJ-587 synthesis (Scheme 1d). Slightly different from the two synergistic Li/Li bimetallic systems mentioned above, the two Li complexes with new coordination modes could also activate the nucleophile and electrophile, respectively, thus promoting the addition reaction synergistically. Furthermore, the two optimal ligands of the synergistic system and the required combinations could be quickly discovered by detailed screening of the ligand library we have established.

To prove our hypothesis, we started to establish the synergistic Li/Li bimetallic system by optimizing suitable ligands. When the reaction was performed at −60 °C without a chiral ligand using LDA as a base (Table 1, entry 1), the reaction afforded the product isomer mixture with a 15% yield and 1:1.2 dr according to the patent method.^9a^ Generally, the most effective and favorable groups in Li^+^ four binding sites were amide and ethereal oxygens due to the fact that both amide and ethereal oxygens are hard bases that interact strongly with Li^+^.^14^ Combined with our previous synergistic Li/Li bimetallic system studies,^11^ we tried to propose a reaction pathway with a synergistic Li/Li bimetallic system mode, as shown in Figure 1. In this mode, two chiral ligands **L**^**1**^ and **L**^**2**^ containing N/O atoms coordinate with Li^+^ to form components of two different chiral Li-complexes on the nucleophile and the electrophile, respectively. In our experience,^11^ a chiral secondary amine ligand **L**^**2**^ reacts with ^*n*^BuLi to form a chiral lithium salt **4***in situ* that behaves as a base to extract a proton from the substrate **1**, forming deprotonated **1** and regenerating the chiral secondary amine **L**^**2**^. Additionally, the chiral lithium salt **4** could coordinate with **L**^**1**^ in solution; thus, a coordination equilibrium exists between **4** and a tridentate complex **5**. According to the resonance theory, the negative charge of the deprotonated **1** could delocalize over the quinoline ring or the benzene ring. Therefore, Li^+^ can be coordinated with N and O atoms on the quinoline ring or F and O atoms on the benzene ring. However, when Li^+^ is coordinated with the N and O atoms of quinoline, an unstable and rigid four-membered ring is formed. Thus, in the proposed possible nucleophile coordination mode, Li^+^ prefers to cocoordinate with F and O atoms to form a five-membered ring complex **6**. Additionally, the excess *in situ* formed chiral lithium salt **4** can coordinate with N and O atoms on the electrophile **2**. The chiral secondary amine **L**^**2**^ regenerated by the deprotonation reaction could co-coordinate with Li^+^ on the electrophile to form a tetradentate complex **7**.

**Figure 1:**
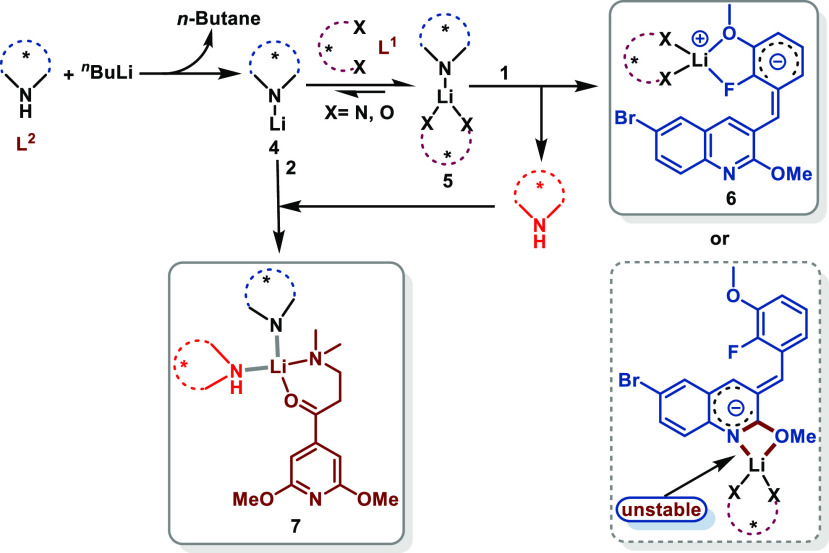
Proposed synergistic Li/Li bimetallic system for the synthesis of TBAJ-587.

**Table 1:**
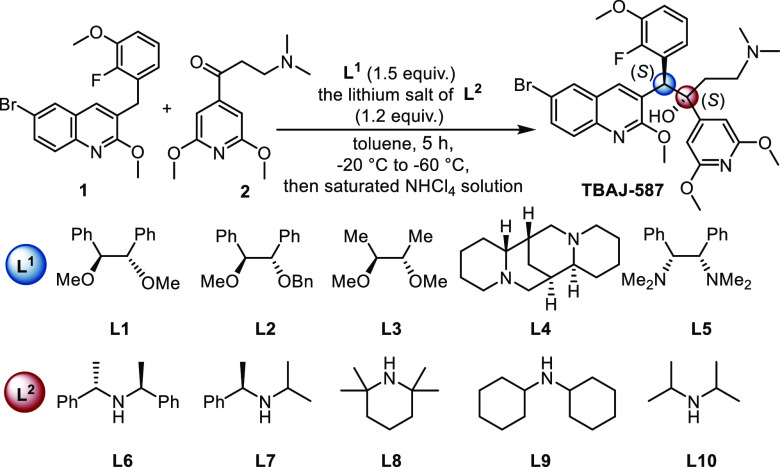
Establishment of the Synergistic Li/Li Bimetallic System

entry^a^	**L**^**1**^	**L**^**2**^**/Li**	yield^b^	dr^c^	er^d^
1		LDA	15%	1.0:1.2	
2	**L1**	**L6**/Li	25%	1.0:1.2	60:40
3	**L2**	**L6**/Li	36%	1.0:1.3	rac
4	**L3**	**L6**/Li	20%	1.0:1.1	rac
5	**L4**	**L6**/Li	50%	1.0:1.3	64:36
6	**L5**	**L6**/Li	21%	1.8:1.0	rac
7	**L4**	**L7**/Li	16%	1.0:1.2	86:14
8	**L4**	**L8**/Li	35%	1.0:1.4	70:30
9	**L4**	**L9**/Li	54%	1.0:1.3	67:33
10	**L4**	**L10**/Li	56%	1.0:1.0	70:30
11	**L4**	LDA	58%	1.0:1.0	72:28
12^e^	**L4**	LDA	40%	1.0:1.0	69:31
13^f^	**L4**	LDA	54%	1.0:1.0	68:32
14^g^	**L4**	LDA	56%	1.0:1.0	69:31
15^h^	**L4**	LDA	56%	1.0:1.0	70:30

a The reaction was conducted with **1** (0.2 mmol), **2** (0.24 mmol), dry toluene (4 mL), LDA (2.0 M in THF/^*n*^heptane/ethylbenzene, 0.24 mmol), **L^1^** (0.30 mmol), and the Li salt of **L^2^** (*in situ* formation by the addition of L^2^ and ^*n*^BuLi (1.6 M in hexane, 0.24 mmol) to the reaction mixture).

b Determined by ^1^H NMR analysis of the crude reaction mixtures, total yield of all isomers of TBAJ-587.

c Determined by ^1^H NMR analysis of the crude reaction mixtures.

d Determined by HPLC analysis.

e The reaction was performed with LDA (0.9 equiv).

f The reaction was performed with LDA (1.5 equiv).

g The reaction was performed with **L4** (1.0 equiv).

h The reaction was performed with **L4** (2.0 equiv).

According to the proposed modes above, we then shifted to optimizing the chiral ligands to form the two distinct catalysts. Initially, a series of chiral diether and diamine ligands **L1**–**L5** in combination with chiral ligand **L6** (typically reported to be used in asymmetric deprotonation lithiation) in toluene were investigated in hope of controlling the enantioselectivity (entries 2–6). Of the five tested ligands, **L4** gave the highest yield of product **3** (50%) and the best enantioselectivity (64:36 er). In order to improve the result, another chiral ligand **L7** for complex **7** was also examined, and a better selectivity was obtained (entry 7, 86:14 er) but with a poor yield. Based on our previous studies, we knew that a simple combination of a chiral ligand and an achiral ligand could enhance both catalytic efficiency and selectivity in the synergistic Li/Li bimetallic system (see the Supporting Information for the comprehensive optimization of the chiral ligands).^11^ Thus, we employed **L8** as the achiral ligand to match **L4**, which formed a triple-coordinated Li complex for the electrophile and provided the best result for the synthesis of TBAJ-876. Yet, no better result for the synthesis of TBAJ-587 was obtained (entry 8). Then, achiral ligands **L9** and **L10** were used to carry out the reaction (entries 9 and 10, respectively). To our delight, diisopropylamine (**L10**) provided the highest yield and moderate selectivity (entry 10, 56% yield, 70:30 er). For ease of operation, the commercial LDA was also investigated (entry 11). Pleasingly, both the yield and er were improved. To further improve the result, we screened the dosage of LDA and **L4**. As anticipated, when the loadings of LDA were reduced, a lower yield was obtained, most probably due to the incomplete lithiation of **1** (entry 12). Additionally, a loss in yield was observed when the amount of LDA was increased due to the generation of the debrominated byproduct (entry 13 vs entry 11). At this time, it occurred to us that after the acid–base reaction between LDA and the nucleophile, the excess LDA could behave as a Lewis acid to activate the electrophile in toluene. The generated diisopropylamine after the acid–base reaction should act as a ligand to occupy the rest of the coordination sites of Li^+^ on the electrophile to form a tetradentate complex. All these results suggested the existence of a synergistic Li/Li bimetallic system. The equivalent amount of LDA was used as a base to react with **1**, and the additional amount of LDA was used as a catalytic species to activate the electrophile **2**. The loadings of **L4** were also evaluated, but the results could not be improved (entries 14 and 15). Many other ligands have also been explored but did not provide any benefits (for details, see the Supporting Information).

With these promising results in hand, we continued to optimize various reaction conditions in order to obtain better results. A series of additives were evaluated, as shown in Table 2. First, when the reaction system was conducted with 4 Å MS (10 mg), surprisingly, compared with the results above (entry 1), both the yield and enantioselectivity were improved (entry 2). Further increasing the dosage of 4 Å MS (molecular sieves) to 20 mg provided higher enantioselectivity (80:20 er) with a satisfactory yield (89%, entry 3). However, the use of more 4 Å MS (50 mg, entry 4) resulted in a lower yield and selectivity. Other additives, such as 3 Å MS, 5 Å MS, and MgSO_4_, were also tested, but no improvements in reaction outcome were observed. After an extensive survey of reaction parameters by varying the solvent, concentration, and temperature (see the Supporting Information), the optimum reaction conditions were obtained as follows: a mixture of **1** (1.0 equiv) and **2** (1.2 equiv) in toluene in the presence of **L4** (1.5 equiv), LDA (1.2 equiv), and 4 Å MS (20 mg for 0.2 mmol of **1**) at −60 °C stirred 5 h.

**Table 2:**
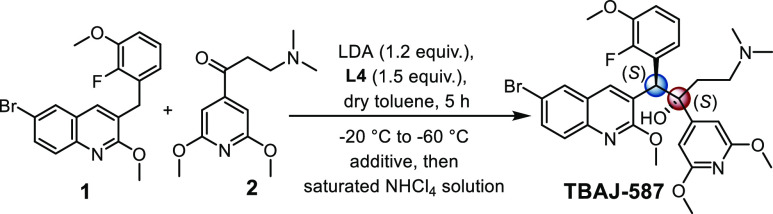
Further Screening of the Reaction Conditions

entry^a^	additive	yield^b^	dr^c^	er^d^
1		58%	1.0:1.0	72:28
2	4 Å MS (10 mg)	73%	1.0:1.0	75:25
3	4 Å MS (20 mg)	89%	1.0:1.0	80:20
4	4 Å MS (50 mg)	86%	1.0:1.0	76:24
5	3 Å MS (20 mg)	66%	1.0:1.0	78:22
6	5 Å MS (20 mg)	64%	1.0:1.0	75:25
7	MgSO_4_ (20 mg)	65%	1.0:1.0	71:29

a The reaction was conducted with **1** (0.2 mmol), **2** (0.24 mmol), dry toluene (4 mL), LDA (2.0 M in THF/^*n*^heptane/ethylbenzene, 0.24 mmol), and **L4** (0.30 mmol).

b Determined by ^1^H NMR analysis of the crude reaction mixtures, total yield of all isomers of TBAJ-587.

c Determined by ^1^H NMR analysis of the crude reaction mixtures.

d Determined by HPLC analysis.

After the establishment of the optimal conditions, the reaction was performed on a 5 g scale smoothly and completed with a 90% yield, 1:1 dr, and 80:20 er (Figure 2a). Then, the obtained diastereoisomers were separated by silica gel column chromatography. Finally, we explored a simple and efficient recrystallization method for obtaining pure product (Figure 2b). TBAJ-587 and its enantiomer (5 g, 80:20 er) were dissolved in the mixed solvent of DCM and ^*i*^PrOH (v/v = 1:7) and then left to stand at ambient temperature for 48 h. The precipitation of solids was observed during this process and their er was determined. Surprisingly, the er of the product in the solution was 99.9:0.1, while it was 60:40 in the solid. The solid was filtered, and the filtrate was evaporated *in vacuo* to give 2.6 g of TBAJ-587 with 99.9:0.1 er (23% yield). The residual solid (2.4 g, 60:40 er) was separated by chiral HPLC resolution to give 1.0 g of TBAJ-587 (12% yield, 99.9:0.1 er). This was combined with the product, which after static settlement in solvent gave a total 3.9 g of TBAJ-587 (35% yield, 99.9:0.1 er). All these results show the potential application of this protocol.

**Figure 2:**
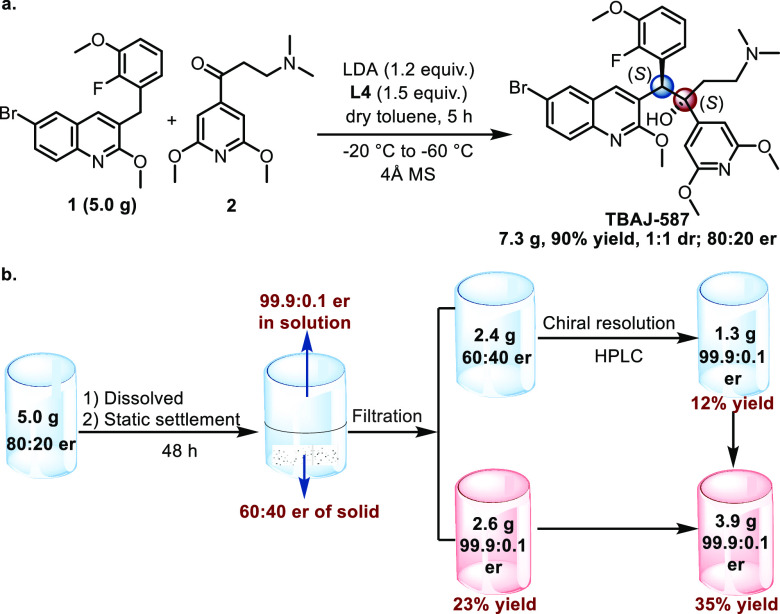
Gram-scale synthesis and separation of TBAJ-587.

Based on the above observations and in combination with previous reports, a possible Li/Li bimetallic system is proposed for the asymmetric synthesis of TBAJ-587, as shown in Scheme 2. Initially, an equivalent of LDA reacts with substrate **1** to yield deprotonated **1** and ^*i*^Pr_2_NH. Then, deprotonated **1** and **L4** coordinate with Li^+^ to produce complex **8**. At the same time, the excess LDA co-coordinates with the generated ^*i*^Pr_2_NH and substrate **2** to form the four-coordinated complex **9**. Then, complex **8** reacts with complex **9** to form intermediate **10**. After a ligand and Li^+^ exchange process, one Li^+^ (blue-marked) and ^*i*^Pr_2_N^–^ are released with ^*i*^Pr_2_NH from **10**. Meanwhile, the other Li^+^ (red-marked) remains, affording intermediate **11**. Finally, the catalytic species LDA regenerated by the free Li^+^ and ^*i*^Pr_2_N^–^ is thus available for the next cycle. After the reaction is quenched by water, intermediate **11** is hydrolyzed to produce product **3** and release **L4**.

**Scheme 2:**
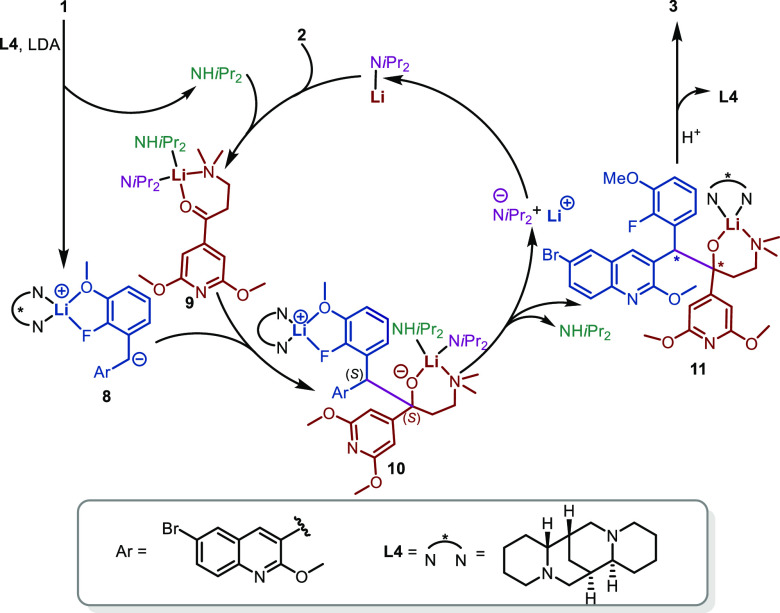
Proposed Mechanism

In conclusion, we have developed a novel synergistic Li/Li bimetallic system for the first stereo- and chemoselective synthesis of the antituberculosis drug TBAJ-587. In the system, two distinct four-coordinated Li complexes formed by the Li^+^ and ligands with the nucleophile and electrophile, respectively, concurrently activate both the nucleophile and the electrophile synergistically. The reaction could be conducted smoothly on a 5 g scale and afforded the product with excellent yield and moderate enantioselectivity. Furthermore, the pure target product could be obtained by simple recrystallization in a suitable solvent.

## Data Availability

The data underlying this study are available in the published article and its Supporting Information.

## Supporting Information Available

General details, asymmetric and gram-scale synthesis of TBAJ-587, and characterization data (PDF)
