# Supplementary material for: Synergistic Li/Li Bimetallic System for the Asymmetric Synthesis of Antituberculosis Drug TBAJ-587
Source: J Org Chem. 2023 May 1;88(11):7601–6. doi: 10.1021/acs.joc.3c00705 (PMC10242759; doi:10.1021/acs.joc.3c00705)
Supplement: Supplementary file 1 — jo3c00705_si_001.pdf [file jo3c00705_si_001.pdf]

Synergistic Li/Li Bimetallic System for the Asymmetric Synthesis of Anti-tuberculosis Drug TBAJ-587

Tanveer Ahmad,[a] Feng Gao,[a] Jing Li,[b] Zhenfeng Zhang,[b] Tao Song,[a] Qianjia Yuan[a] and Wanbin Zhang*[a][b]

a School of Chemistry and Chemical Engineering, Shanghai Jiao Tong University, 800 Dongchuan Road, Shanghai 200240, P. R. China

b Shanghai Key Laboratory for Molecular Engineering of Chiral Drugs, School of Pharmacy, Shanghai Jiao Tong University, 800 Dongchuan Road, Shanghai 200240, P. R. China

Fax: (+)86-21-54743265; Phone: (+)86-21-34207176; E-mail: wanbin@sjtu.edu.cn

Contents

1. General Details.....S2

2. Asymmetric Synthesis of TBAJ-587.....S2

3. Gram-scale Synthesis of TBAJ-587.....S7

4. Characterization Data.....S8

5. Reference.....S13

1. General Details

All reactions were conducted in flame-dried glassware under an atmosphere of argon/ nitrogen, and the workup was carried out in air unless otherwise noted. All the reagents and super dry solvents were purchased from commercial suppliers and used without further purification. The reactions were conducted at -20 °C to -60 °C in a low temperature cooling circulation machine. Reactions were monitored through analytical thin layer chromatography (TLC) (SiO_2 60 F-254 plates). Flash column chromatography (FCC) was performed by using 100–200 mesh silica gel (SiO_2 60). ^{1}H NMR spectra were recorded on a Brucker Ascend™ 400 (400 MHz) and Brucker Ascend™ 500 (500 MHz). ^{13}C NMR spectra were recorded on a Brucker Ascend™ 400 (100 MHz) and Brucker Ascend™ 500 (125 MHz). Matrix-Assisted Laser Desorption/Ionization Time of Flight Mass Spectrometry (MALDI-TOF MS) was performed on a Fourier-transform mass spectrometer at the Instrumental Analysis Center of Shanghai Jiao Tong University. Enantioselectivity was measured by a high performance liquid chromatography (HPLC) using Daicel Chiralcel AD-H columns with *n*-hexane/*i*-PrOH as eluent.

The starting materials **1**[1], **2**[1] and ligand **L4**[2] are known compounds, which have been commercially available and were used directly without further purification. The ligands **L1**, **L4-L10**, **L14**, **L16-L39** were commercially available and were used directly without further purification. The ligands **L2**, **L3**[3], **L11-L12**[4], **L13**[5], and **L15**, **L17**[4] were synthesized according to the reported literatures.

2. Asymmetric Synthesis of TBAJ-587

2.1. General experimental procedure for the asymmetric synthesis of TBAJ-587

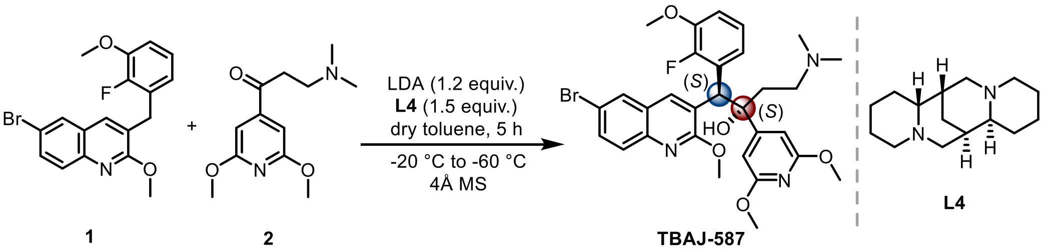

1 **2** **TBAJ-587**

All glassware were pre-dried in the oven, and further flame dried by heating gun before using. To the solution of **1** (75.0 mg, 0.2 mmol, 1.0 equiv.), **L4** (70.3 mg, 0.3 mmol, 1.5 equiv.) and 4Å MS (20 mg) in dry toluene (2 mL) stirring at -20 °C for 5 minutes, 0.13 mL of 2.0 M lithium diisopropylamide (LDA) (0.24 mmol, 1.2 equiv.) in THF/*n*-heptane/ethylbenzene was added dropwise at -20 °C under the atmosphere of argon. The reaction mixture was further stirred at the same temperature. After stirring for 1 hour, a solution of **2** (57.1 mg, 0.24 mmol, 1.2 equiv.) in dry toluene (1.5 mL) was added *via* a double necked needle (1-2 minutes) and the resulting mixture was stirred at – 60 °C for 4 h. The reaction was quenched with 2-3 mL of saturated NH_4Cl solution and extracted with ethyl acetate (3 × 5.0 mL). The organic layer was dried over anhydrous Na_2SO_4 and

concentrated *in vacuo* to give a light brownish yellow oil. The dr of crude product was analyzed by ^{1}H NMR (dr = [(*S*,*S*)+(*R*,*R*)] : [(*S*,*R*)+(*R*,*S*)]). After that, the crude product was purified by FCC using petroleum ether/ethyl acetate (10/1–10/10) and MeOH/CH_2Cl_2 as eluent to separate the diastereomers, the R_f of the desired product is 0.35 (the desired isomer, petroleum ether/ethyl acetate = 1/1), and the er [(*S*,*S*):(*R*,*R*)] was determined by HPLC.

2.2. Screening of reaction conditions

Scheme S1. Optimization of different chiral ligands

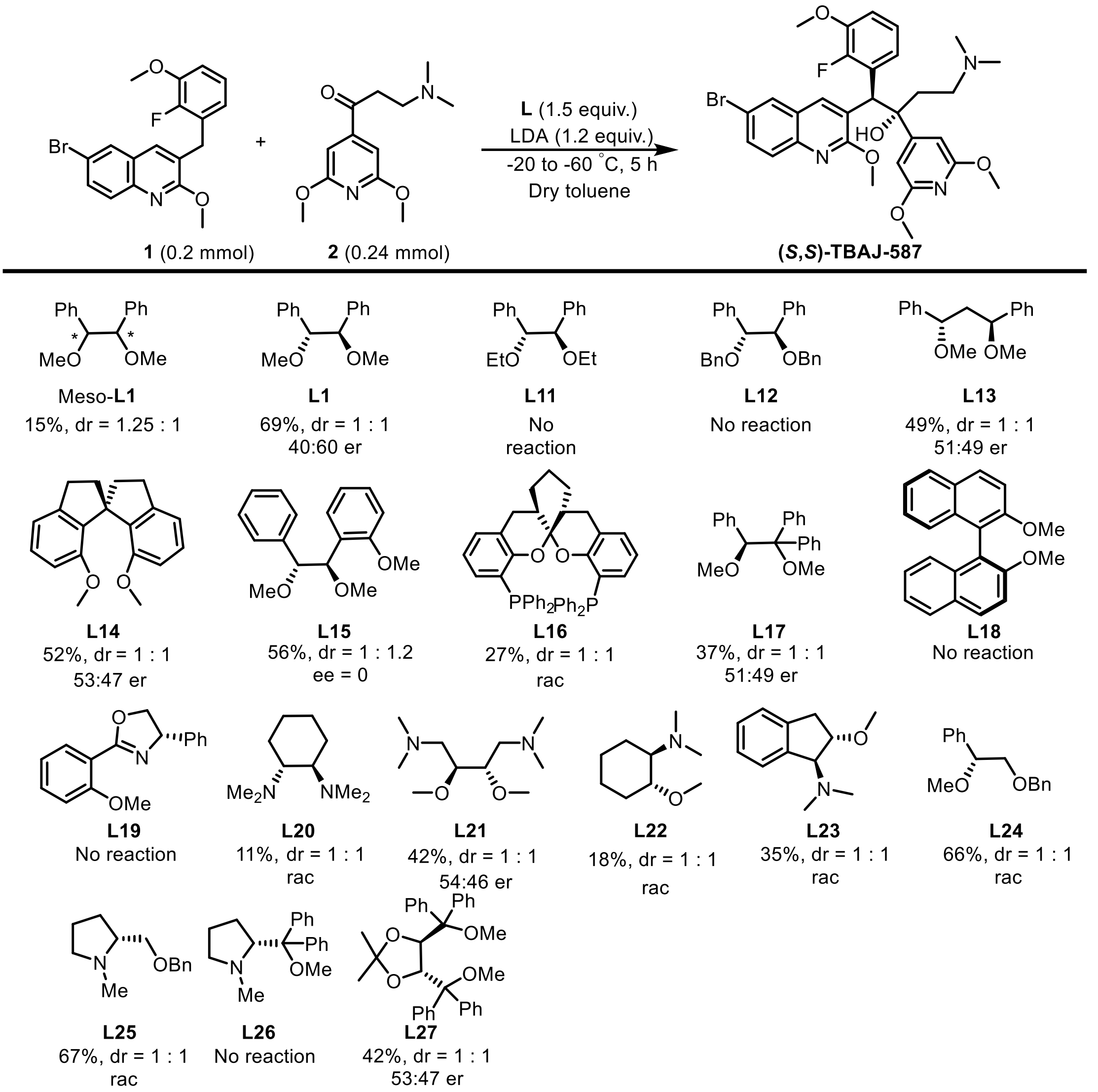

Isolated yield of all four diastereomers; dr was determined by the crude ^{1}H NMR, dr = [(*S*,*S*)+(*R*,*R*)] : [(*S*,*R*)+(*R*,*S*)]; er was determined by HPLC.

Scheme S2. Optimization of different chiral amine ligands to match L4

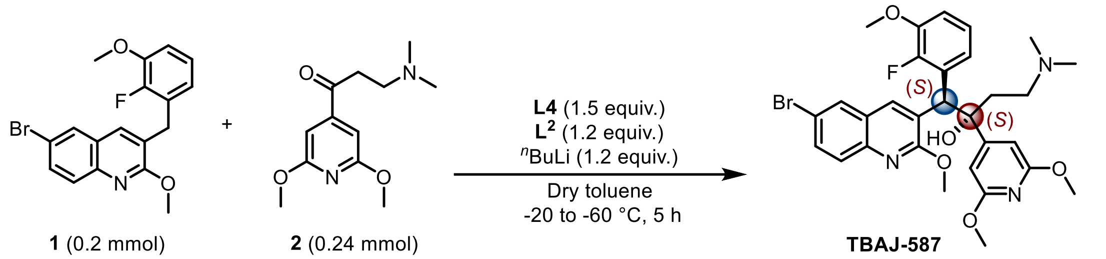

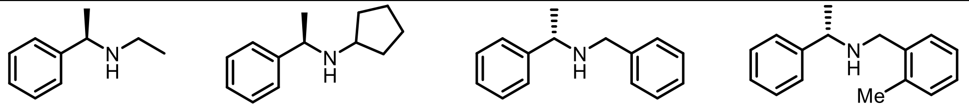

L28	L29	L30	L31
30%, dr = 1.1 : 1<br>60:40 er	31%, dr = 1 : 1<br>76:24 er	34%, dr = 1.2 : 1<br>32:68 er	19%, dr = 1 : 1<br>49:51 er

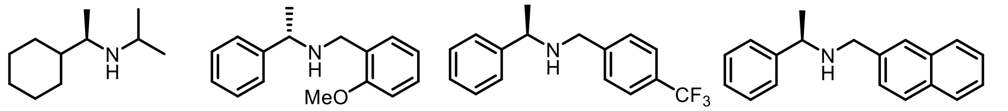

L32	L33	L34	L35
22%, dr = 1 : 1.3<br>64:36 er	No Reaction	29%, dr = 1.1 : 1<br>65:35 er	65%, dr = 1 : 1.3<br>57:43 er

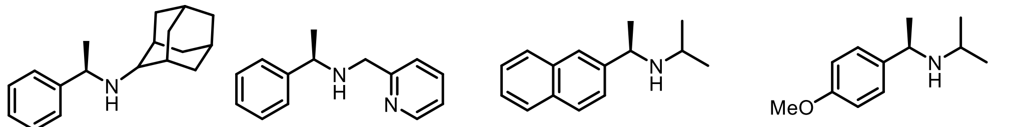

L36	L37	L38	L39
33%, dr = 1 : 1.8<br>66:34 er	Trace	20%, dr = 1 : 1<br>58:42 er	35%, dr = 1 : 1<br>80:20 er

Isolated yield of all four diastereomers; dr was determined by the crude ^{1}H NMR, dr = [(*S,S*)+(*R,R*)] : [(*S,R*)+(*R,S*)]; er was determined by HPLC.

Table S1. Optimization of the base

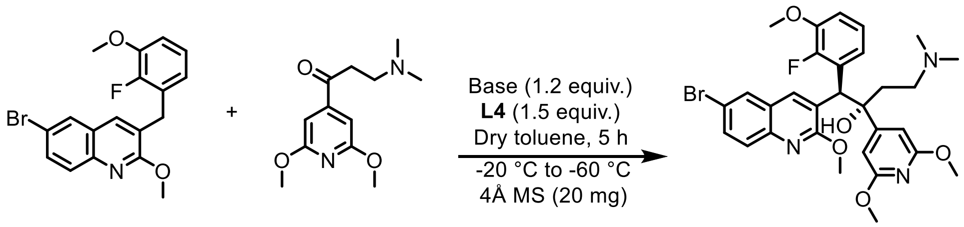

1 2 (*S*,*S*)-TBAJ-587

entry	base	yield (%)[a]	dr[b]	er[c]
1	KHMDS	trace	-	-
2	NaHMDS	-	-	-
3	LiHMDS	-	-	-
4	LiTMP	70	1:1	71:29
5	$LiNCy_2$	38	1.3:1	79:21
6	*n*-BuLi	by product	-	-
7	LDA	80	1:1	80:20

[a]Isolated yields of all four diastereomers; [b]dr was determined by the crude ^{1}H NMR, dr = [(*S*,*S*)+(*R*,*R*)] : [(*S*,*R*)+(*R*,*S*)]; [c]er was determined by HPLC.

Table S2. Optimization of the temperature

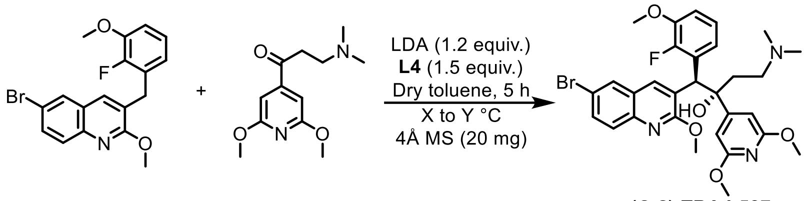

entry	temp. (°C)	yield (%)[a]	dr[b]	er[c]
1	0 to -20 to -60	trace	-	-
2	-10 to -20 to -60	45	1:1.2	77:23
3	-20 to 0 to -20 to -60	50	1:1.2	72:28
4	-20 to r.t. to -20 to -60	46	1:1.3	72:28
5	-20 to -20 to -60	80	1:1	80:20
7	-10 to -10 to -60	trace	-	-
8	-20 to -78	78	1.1:1	79:21

[a]Isolated yields of all four diastereomers; [b]dr was determined by the crude ^{1}H NMR, dr = [(*S*,*S*)+(*R*,*R*)] : [(*S*,*R*)+(*R*,*S*)]; [c] er was determined by HPLC.

Table S3. Optimization of the solvent

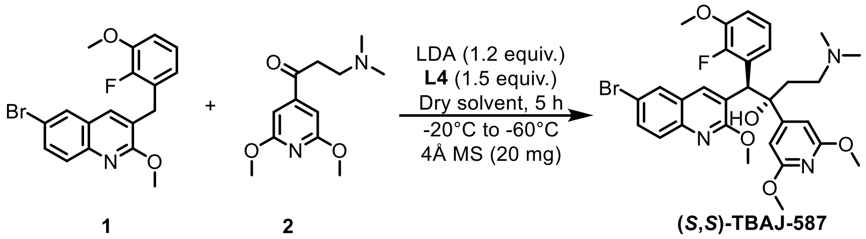

entry	solvent	temp. (°C)	yield (%)[a]	dr[b]	er[c]
1	Toluene	-20 to -60	80	1:1	80:20
2	*o*-xylene	-20 to -20	70	1:1	80:20
2	Mesitylene	-20 to -20	46	1:1	67:33
3	CCl_4	-20 to -20	trace	-	-
4	Anisol	-20 to -30	67	1:1	60:40
5	*m*-xylene	-20 to -40	66	1:1	77:23
6	PhCl	-20 to -40	trace	-	-
7	*t*-butyl benzene	-20 to -40	35	1:1	68:32
8	PhF	-20 to -60	trace	-	-
9	MTBE	-20 to -60	38	1:1	70:30
10	THF	-20 to -60	46	1:1.5	53:47
11	1,3-dioxolane	-20 to -60	33	1:1.4	52:48
12	Toluene : *o*-xylene	-20 to -35	66	1:1	75:25

[a]Isolated yields of all four diastereomers; [b]dr was determined by the crude ^{1}H NMR, dr = [(*S*,*S*)+(*R*,*R*)] : [(*S*,*R*)+(*R*,*S*)]; [c] er was determined by HPLC.

Table S4. Optimization of the solvent concentration

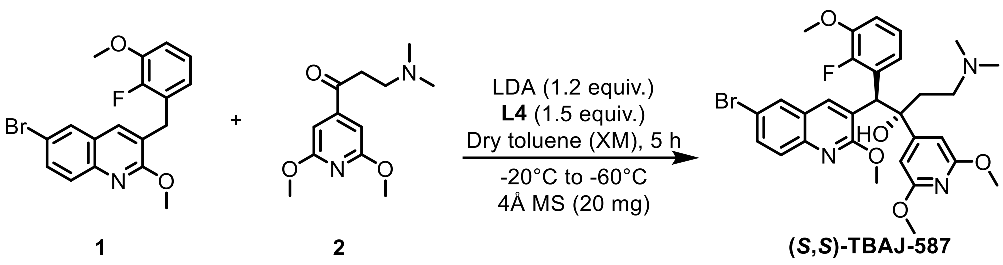

entry	concentration (XM)	yield (%)[a]	dr[b]	er (%)[c]
1	0.03 M	78	1.1 : 1	78:22
2	0.05 M	80	1 : 1	80:20
3	0.07 M	70	1 : 1	79:21

[a]Isolated yields of all four diastereomers; [b]dr was determined by the crude ^{1}H NMR, dr = [(*S*,*S*)+(*R*,*R*)] : [(*S*,*R*)+(*R*,*S*)]; [c] er was determined by HPLC.

Table S5. Optimization of the different additives

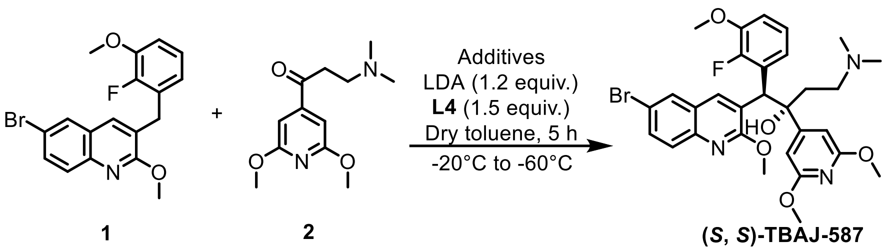

entry	additives	yield (%)[a]	dr[b]	er[c]
1	18-crown-6 (0.5 eq.)	32	1:1.4	53:47
2	THF (0.5 eq.)	60	1:1.2	67:33
3	HMPA (0.5 eq.)	40	1:1.3	rac.
4	TMEDA (0.5 eq.)	65	1:1.5	69:31
5	DMPU (0.5 eq.)	67	1:1.9	rac.
6	NEt_3 (0.5 eq.)	70	1:1.1	70:30
7	$MgSO_4$ (0.5 eq.)	65	1:1	71:29
8	LiOAc (0.5 eq.)	70	1:1	72:28
9	LiI (0.5 eq.)	64	1:1	72:28
10	$LiClO_4$ (0.2 eq.)	70	1:1	74:26
11	$Mg(ClO_4)_2$ (0.2 eq.)	70	1:1	69:31
12	3Å MS (20 mg)	66	1:1	78:22
13	4Å MS (20 mg)	89	1:1	80:20

[a]Isolated yields of all four diastereomers; [b]dr was determined by the crude ^{1}H NMR, dr = [(*S*,*S*)+(*R*,*R*)] : [(*S*,*R*)+(*R*,*S*)]; [c]er was determined by HPLC.

Table S6. Optimization of the reaction scale

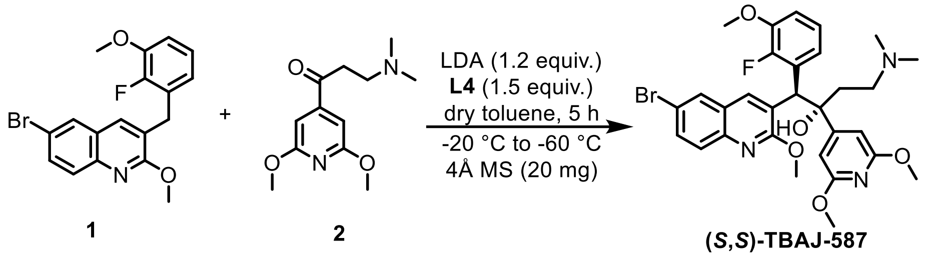

entry	reaction scale	yield (%)[a]	dr[b]	er[c]
1	0.2 mmol	80	1:1	80:20
2	0.4 mmol	78	1:1	79:21
3	0.8 mmol	76	1:1	79:21

[a]Isolated yields of all four diastereomers; [b]dr was determined by the crude ^{1}H NMR, dr = [(*S*,*S*)+(*R*,*R*)] : [(*S*,*R*)+(*R*,*S*)]; [c]er was determined by HPLC.

3. Gram-scale Synthesis of TBAJ-587

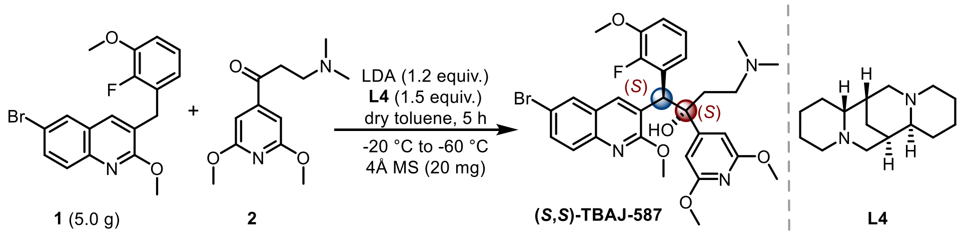

To the solution of **1** (5.0 g, 13.3 mmol, 1.0 equiv.), **L4** (4.7 g, 19.9 mmol, 1.5 equiv.) and 4Å MS (1.3 g) in dry toluene (135 mL) stirred at -20 °C for 5 to 10 minutes, 8.6 mL of 2.0 M lithium diisopropylamide (LDA) (17.3 mmol, 1.3 equiv.) in THF/*n*-heptane/ethylbenzene was added dropwise at -20 °C in the atmosphere of Ar. The reaction mixture was further stirred for more than 1 h at the same temperature. Then, a solution of **2** (3.8 g, 16.0 mmol, 1.2 equiv.) in dry toluene (35 mL) was added dropwise over 5 minutes *via* a double necked needle and the resulting solution was stirred at -60 °C for 4 h. The reaction was quenched with 50 mL of saturated NH_4Cl solution and extracted with ethyl acetate (3 × 100 mL). The organic layer was dried over anhydrous Na_2SO_4 and concentrated *in vacuo* to give a light brownish yellow oil. The crude product was analyzed by ^{1}H NMR and dr was determined (dr = [(*S*,*S*)+(*R*,*R*)] : [(*S*,*R*)+(*R*,*S*)]). After that, the crude product was purified by FCC using petroleum ether/ethyl acetate (10/1–10/10) and MeOH/CH_2Cl_2 as eluent to afford all the diastereomers, R_f = 0.35 (the desired isomer, petroleum ether/ethyl acetate = 1/1). The desired product was obtained as a white solid (7.3 g, 90% yield). HPLC (ChiralPAK AD-H, *n*-hexane/*i*-PrOH = 90/10, UV = 254 nm, flow rate = 1.0 mL/min), t_{R1} = 3.968 min (major) and t_{R2} =

8.842 min (minor), er = 80:20. **^{1}H NMR** (400 MHz, $CDCl_3$): δ 8.40 (s, 1H), 7.82 (d, J = 1.6 Hz, 1H), 7.68-7.63 (m, 2H), 7.56 (dd, J = 9.2, 2.0 Hz, 1H), 6.85 (t, J = 8.0 Hz, 1H), 6.62 (t, J = 8.0 Hz, 1H), 6.52 (s, 2H), 5.40 (s, 1H), 4.15 (s, 3H), 3.87 (s, 6H), 3.69 (s, 3H), 2.35 (t, J = 10.8 Hz, 1H), 2.04 (s, 6H), 1.93 (t, J = 14.0 Hz, 2H), 1.77 (d, J = 14.4 Hz, 1H).

For the undesired diastereomer (*S*,*R*)-**3** or (*R*,*S*)-**3**, HPLC (ChiralPAK AD-H, *n*-hexane/*i*-PrOH = 97/3, UV = 254 nm, flow rate = 1.0 mL/min), t_{R1} = 5.676 min (minor) and t_{R2} = 6.735 min (major); **^{1}H NMR** (400 MHz, $CDCl_3$): δ 8.67 (s, 1H), 7.80 (d, J = 0.8 Hz, 1H), 7.56-7.50 (m, 2H), 7.04 (t, J = 6.4 Hz, 1H), 6.97 (t, J = 8.0 Hz, 1H), 6.85 (t, J = 8.0 Hz, 1H), 6.53 (s, 2H), 5.33 (s, 1H), 3.90 (d, J = 8 Hz, 6H), 3.82 (s, 6H), 2.04 (s, 6H), 2.21-2.02 (m, 4H).

4. Characterization Data

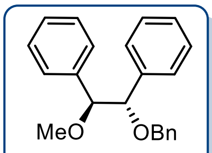

L2

Colorless oil. ^{1}H NMR (500 MHz, $CDCl_3$) δ 7.32-7.25 (m, 5H), 7.22-7.19 (m, 6H), 7.11-7.10 (m, 2H), 7.07-7.05 (m, 2H), 4.57 (d, J = 12.0 Hz, 1H), 4.54 (d, J = 6.5 Hz, 1H), 4.41 (d, J = 7.5 Hz, 1H), 4.37 (d, J = 12.5 Hz, 1H), 3.31 (s, 3H); ^{13}C NMR (500 MHz, $CDCl_3$) δ 138.61, 138.51, 138.44, 128.24, 128.02, 127.86, 127.79, 127.62, 127.61, 127.52, 127.34, 87.60, 84.92, 70.83, 57.53; HR-MS (ESI): m/z= 318.1620, calcd. for $C_{12}H_{22}O_2$ $[M+Na]^+$: 341.1517, found 341.1513.

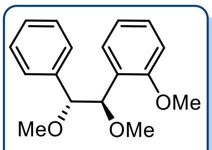

L15

White solid. ^{1}H NMR (500 MHz, $CDCl_3$) δ 7.23-7.20 (m, 4H), 7.09-7.06 (m, 2H), 6.98 (dd, J = 6.0 Hz, 1.5 Hz, 1H), 6.85-6.82 (m, 2H), 5.00 (d, J = 5.0 Hz, 1H), 4.40 (d, J = 5.0 Hz, 1H), 3.70 (s, 3H), 3.26 (s, 3H), 3.23 (s, 3H); ^{13}C NMR (500 MHz, $CDCl_3$) δ 157.43, 137.96, 128.41, 128.38, 128.36, 127,41, 127.37, 126.37, 120.24, 110.10, 85.75, 79.71, 57.39, 57.20, 55.38. HR-MS (ESI): m/z= 272.1412, calcd. for $C_{17}H_{21}O_3$ $[M+Na]^+$: 295.1310, found 295.1303.

4.1. NMR Spectral Data

1-(6-bromo-2-methoxyquinolin-3-yl)-2-(2,6-dimethoxypyridin-4-yl)-4-(dimethylamino)-1-(2-fluoro-3-methoxyphenyl)butan-2-ol

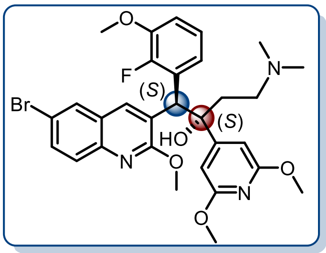

The dr was determined by integration the signal of the proton appeared at 5.40 ppm (desired) and 5.33 ppm (undesired) in the crude ^{1}HNMR spectrum. The enantioselectivity (er) [(*S,S*)/(*R,R*)-diastereomers] was measured by HPLC using chiral column AD-H.

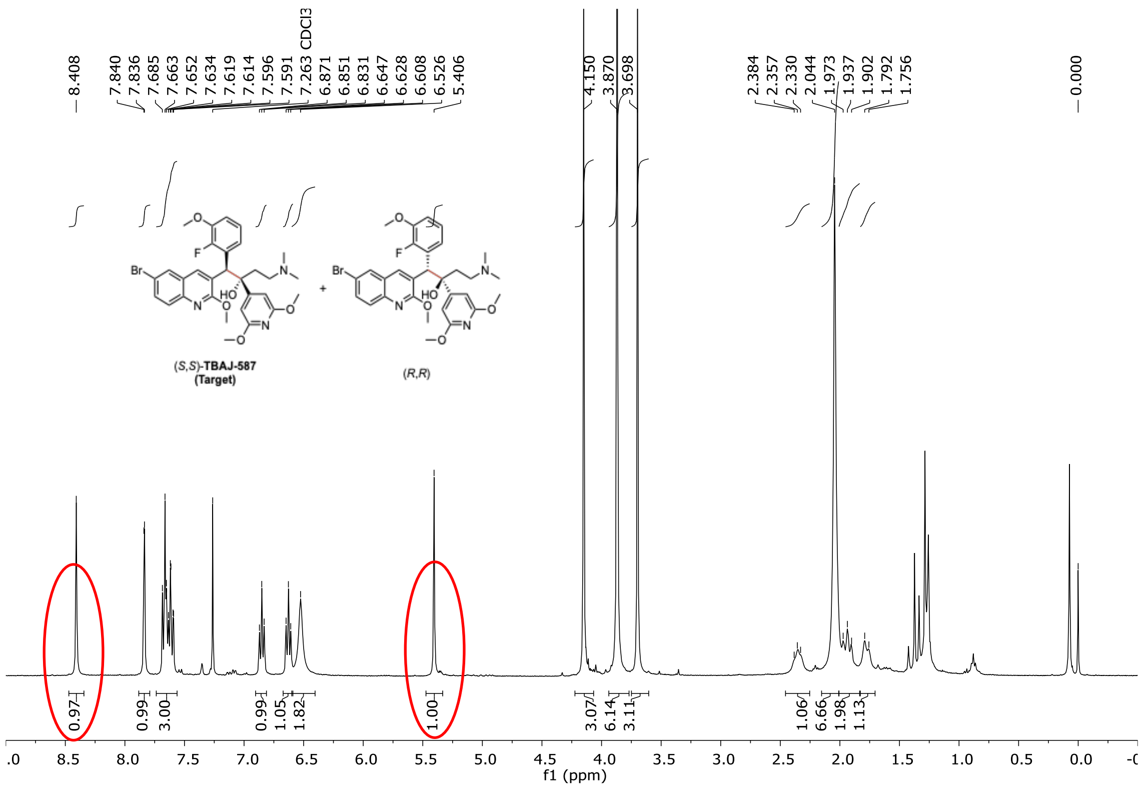

Figure S1. ^{1}H NMR Spectrum (400 MHz, $CDCl_3$) of (*S,S*)/(*R,R*)-TBAJ-587.

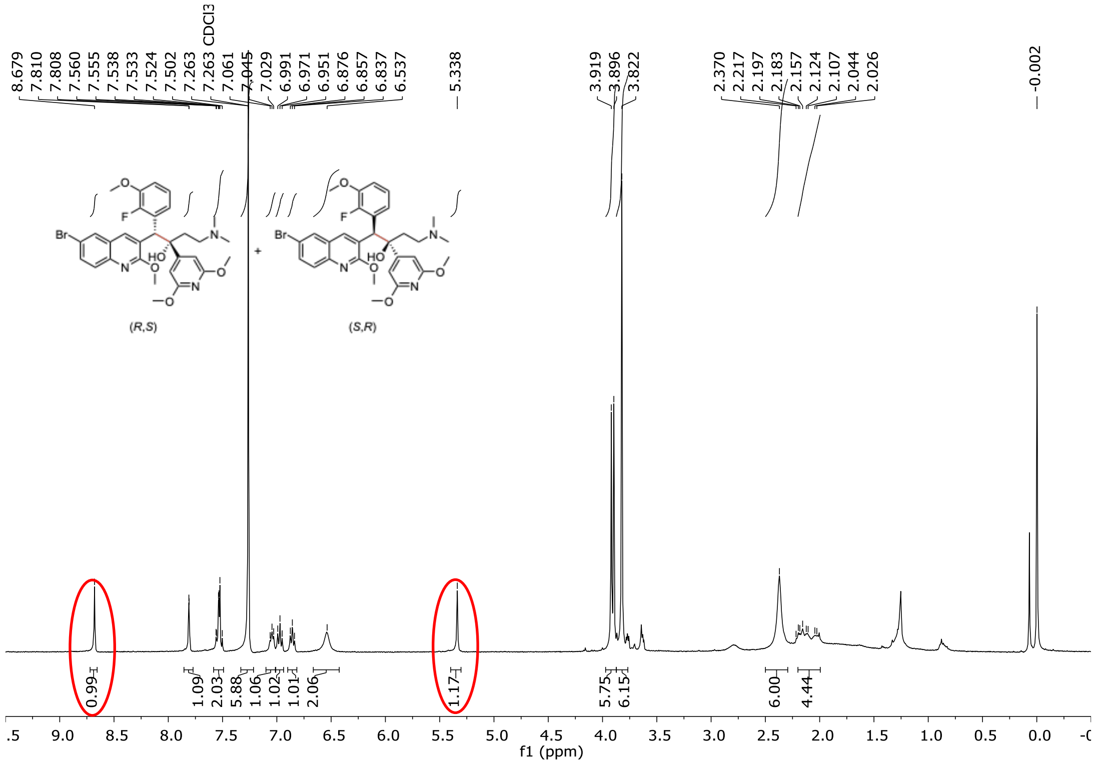

Figure S2. ^{1}H NMR Spectrum (400 MHz, $CDCl_3$) of (*R,S*)/(*S,R*)-TBAJ-587.

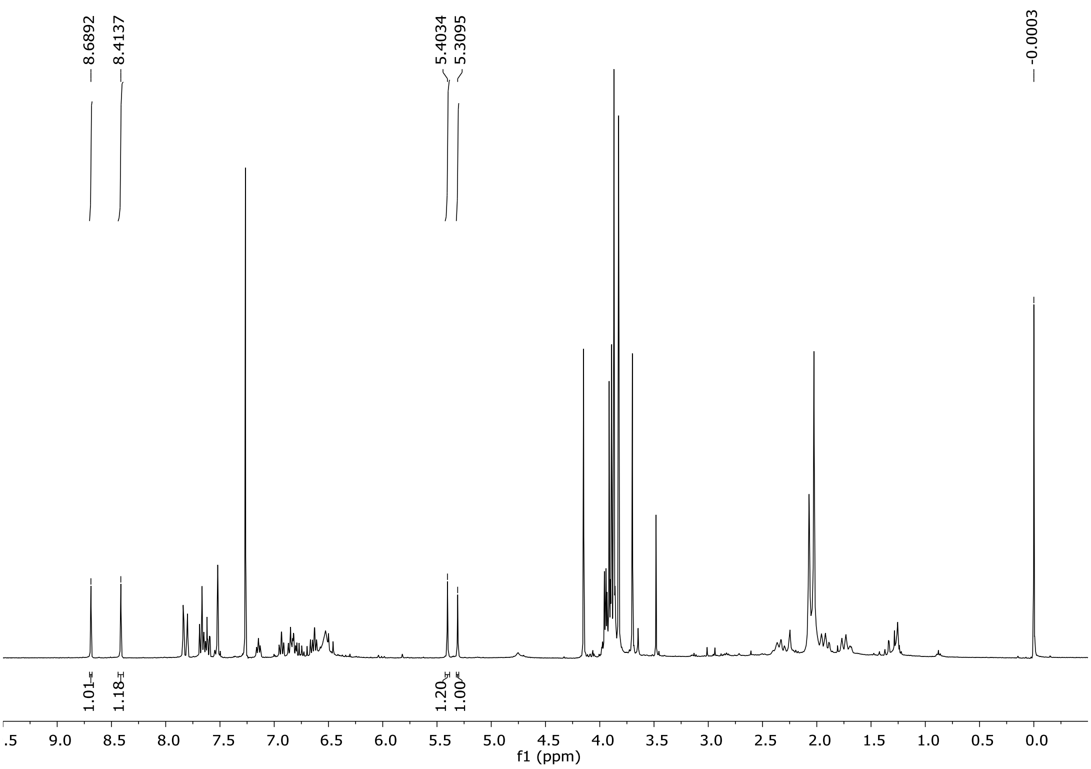

Figure S3. ^{1}H NMR Spectrum (400 MHz, $CDCl_3$) of a crude mixture (1.2:1 dr).

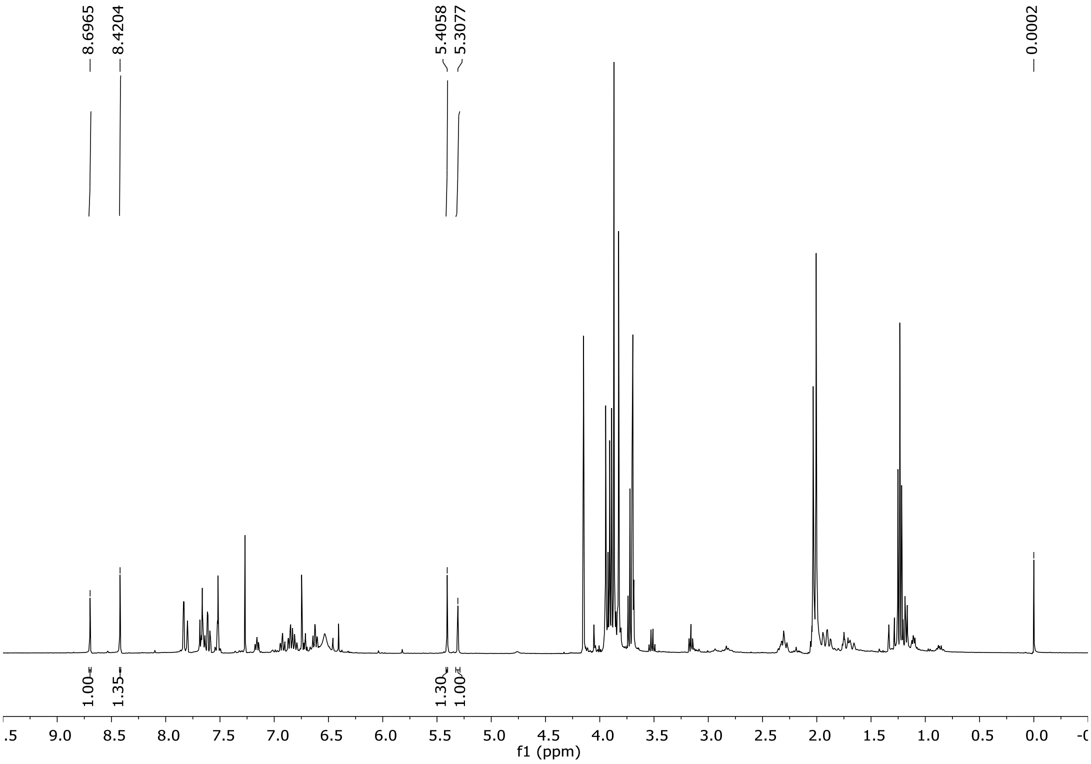

Figure S4. ^{1}H NMR Spectrum (400 MHz, $CDCl_3$) of a crude mixture (1.3:1 dr).

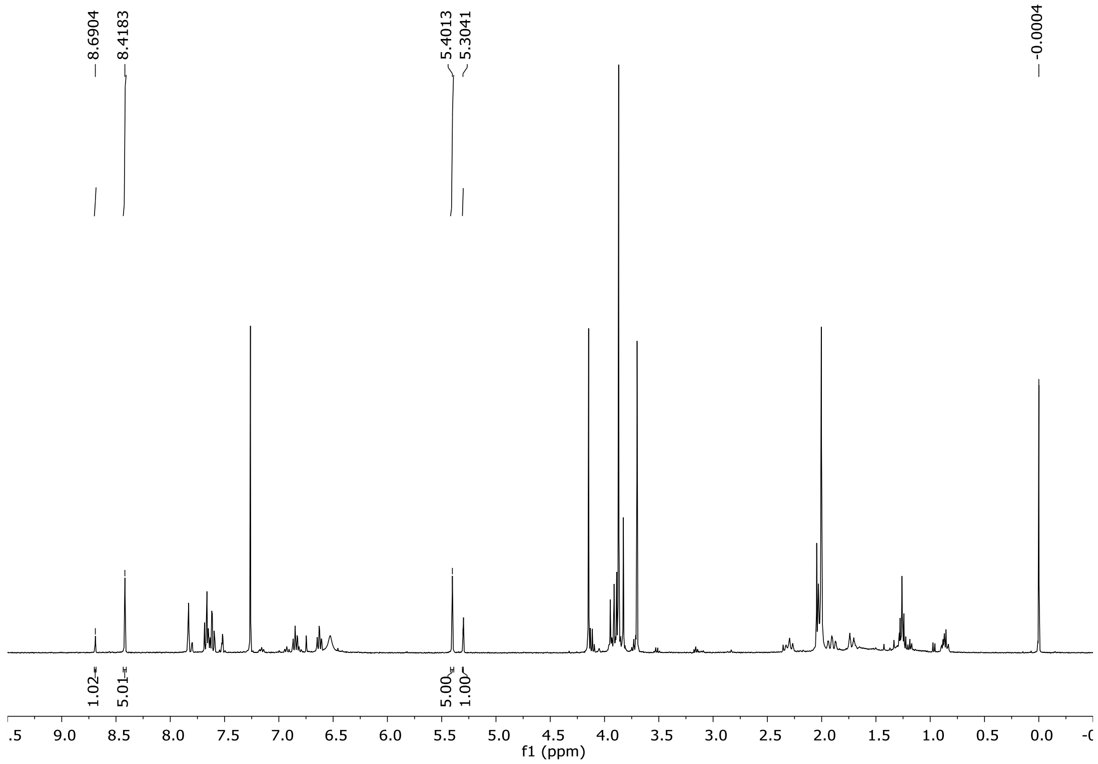

Figure S5. ^{1}H NMR Spectrum (400 MHz, $CDCl_3$) of a crude mixture [(*S*,*S*)+(*R*,*R*)] : [(*S*,*R*)+(*R*,*S*)] (5:1 dr).

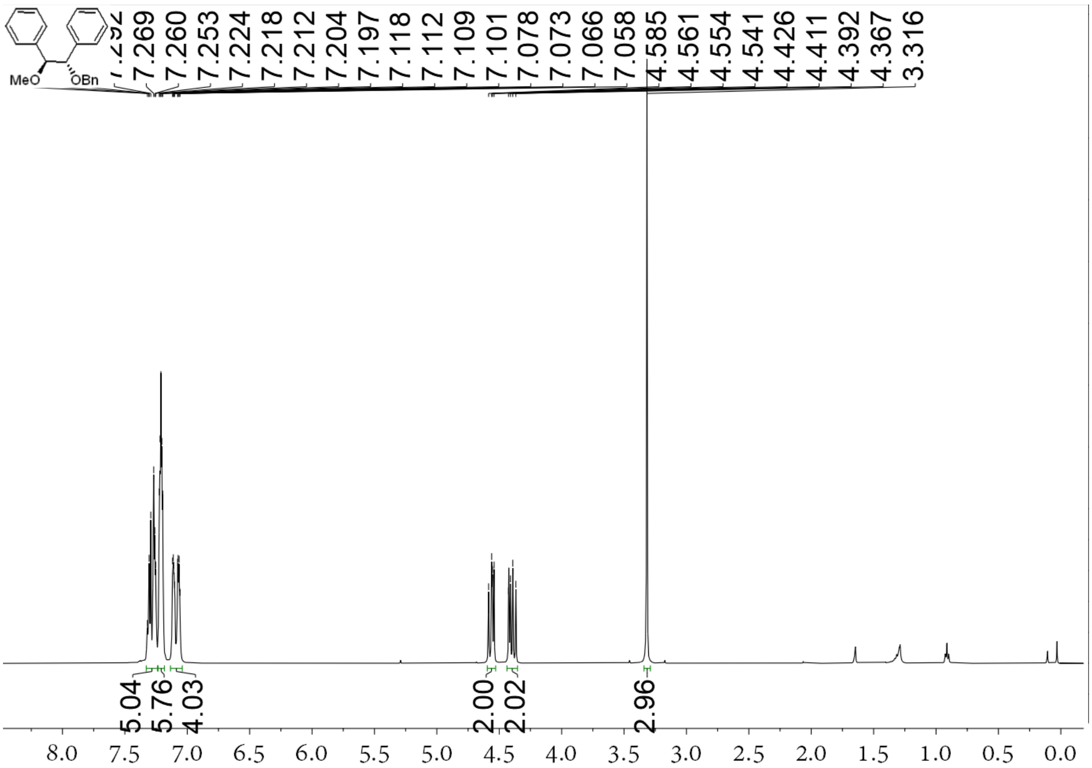

Figure S6. ^{1}H NMR Spectrum (500 MHz, $CDCl_3$) of **L2**

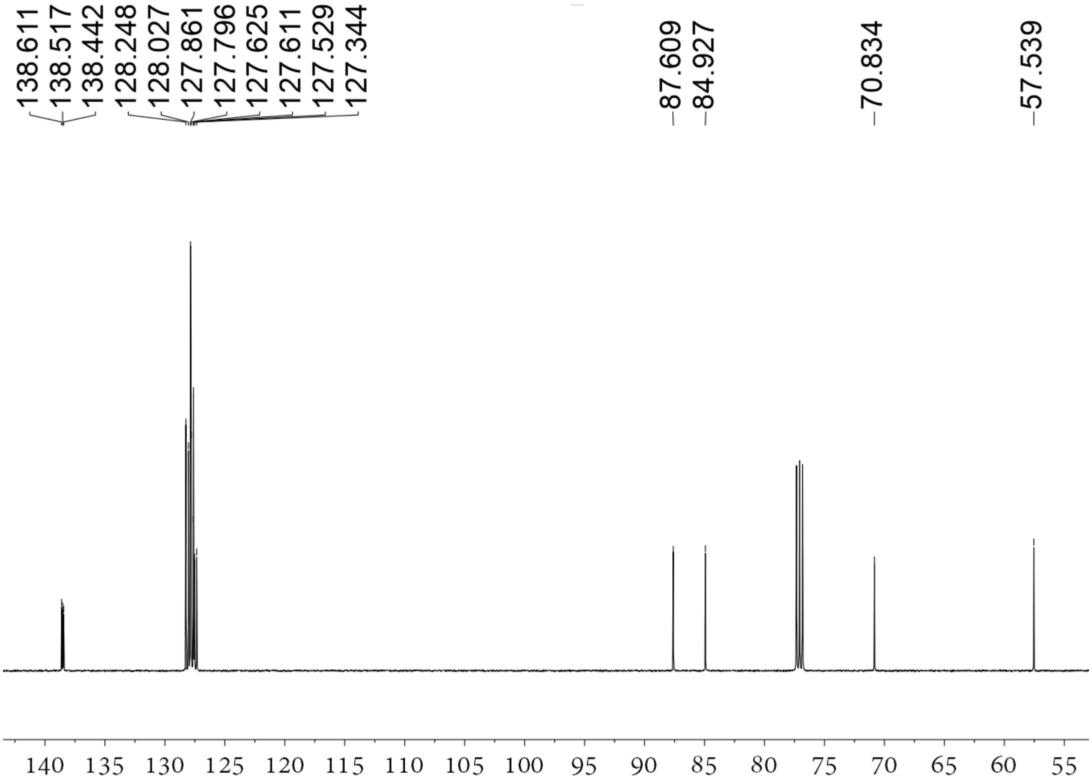

Figure S7. ^{13}C NMR Spectrum (500 MHz, $CDCl_3$) of **L2**

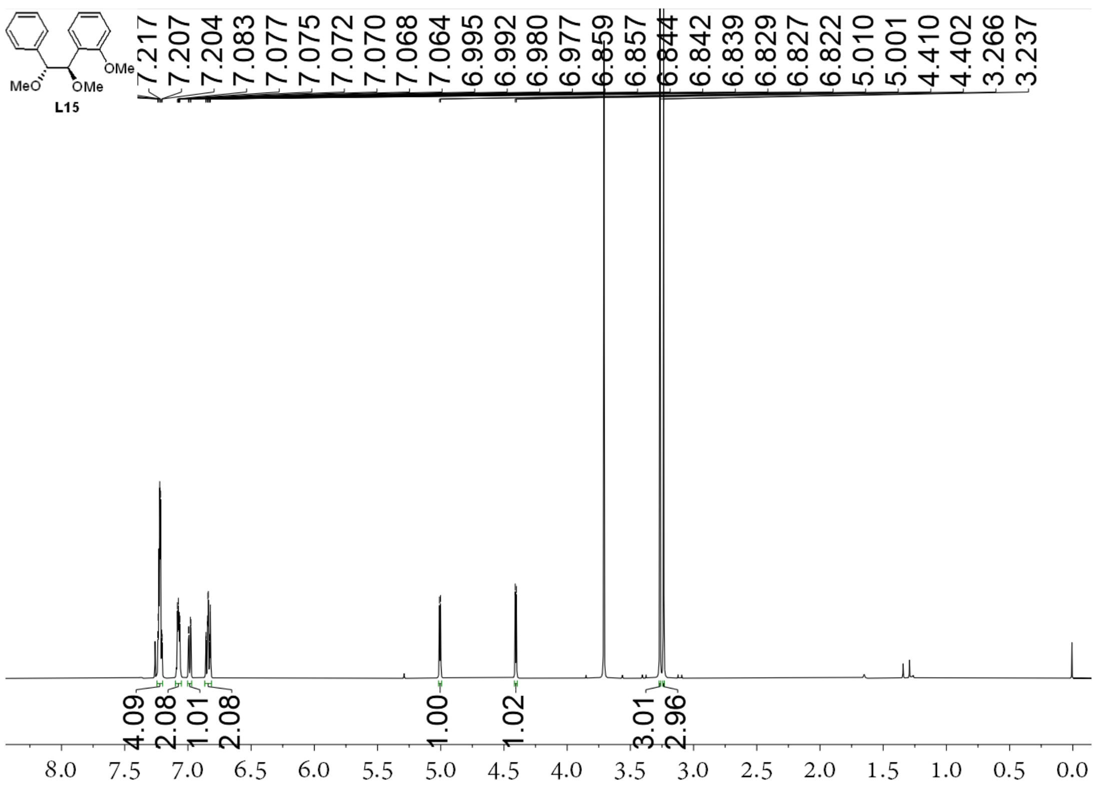

Figure S8. ^{1}H NMR Spectrum (500 MHz, $CDCl_3$) of **L15**

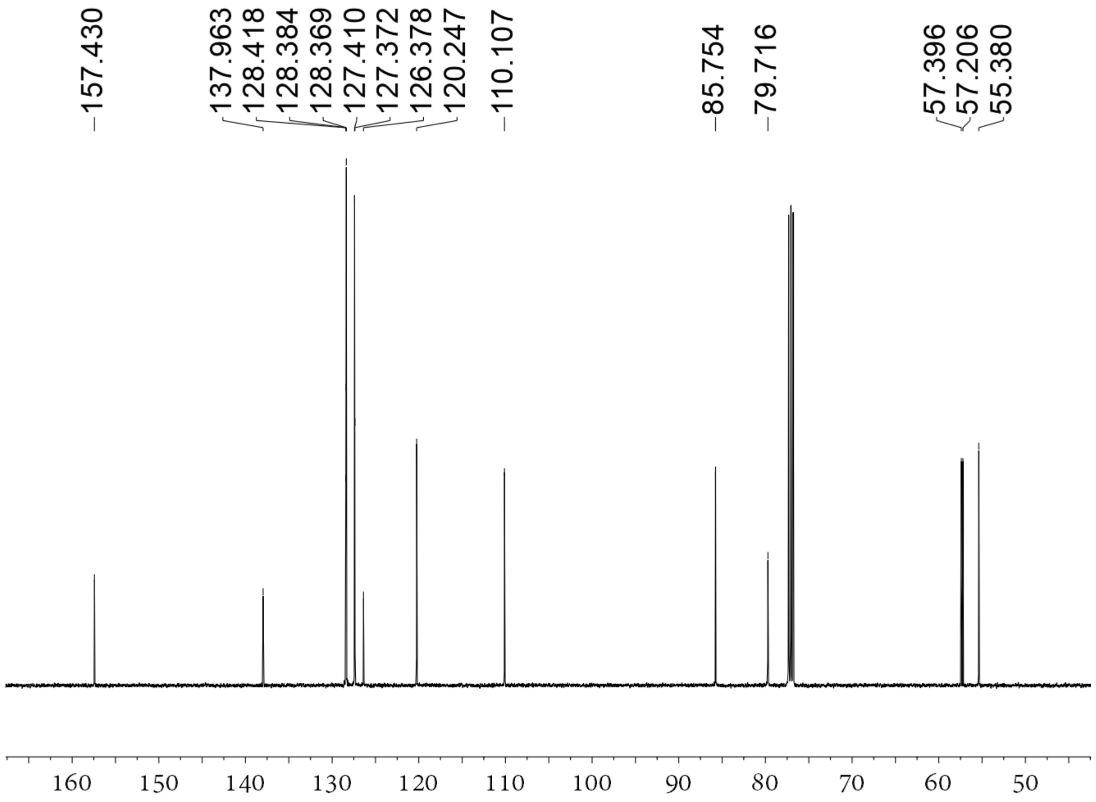

Figure S9. ^{13}C NMR Spectrum (500 MHz, $CDCl_3$) of **L15**

4.2. HPLC Spectral Data

Spectra of (*S,S*)/(*R,R*)-diastereomers:

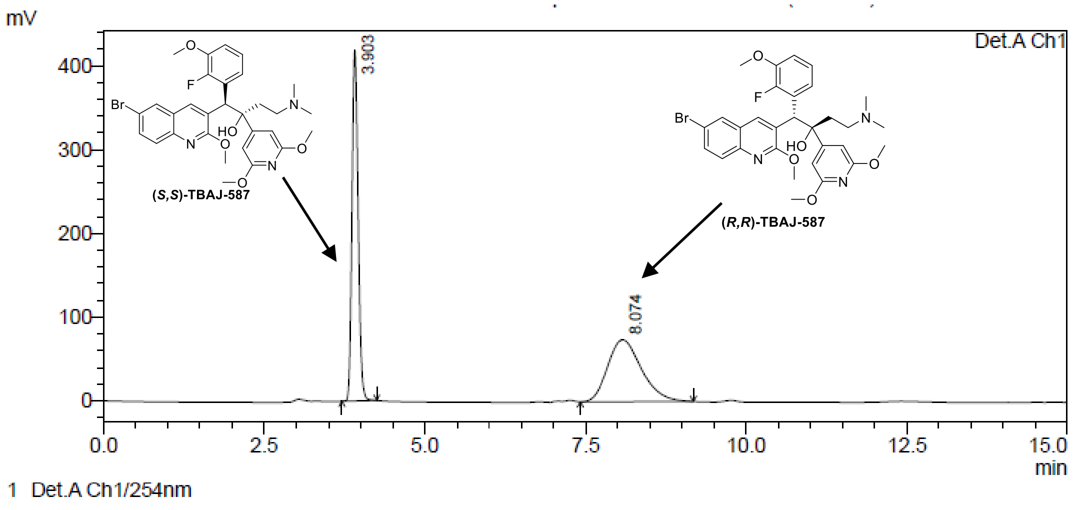

PeakTable

Detector A Ch1 254nm

Peak#	Ret. Time	Area	Height	Area %	Height %
1	3.903	2778369	419107	50.556	84.956
2	8.074	2717279	74215	49.444	15.044
Total		5495649	493322	100.000	100.000

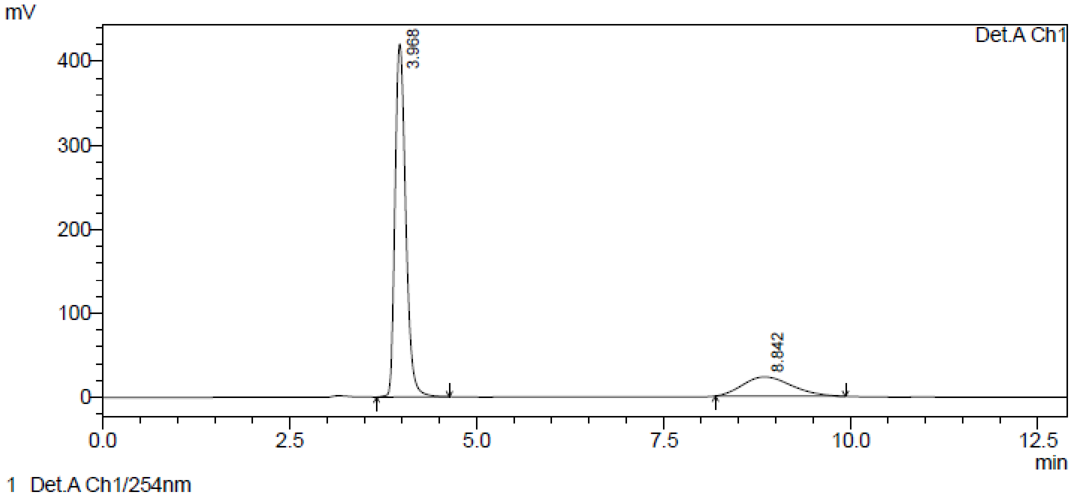

PeakTable

Detector A Ch1 254nm

Peak#	Ret. Time	Area	Height	Area %	Height %
1	3.968	4163503	419941	80.124	94.848
2	8.842	1032812	22809	19.876	5.152
Total		5196315	442750	100.000	100.000

AD-H, 1.0 mL/min, *n*-hexane/*i*-PrOH = 90 : 10, 254 nm, 80:20 er

Figure S10. HPLC spectrum of [(*S,S*)/(*R,R*)-diastereomers]

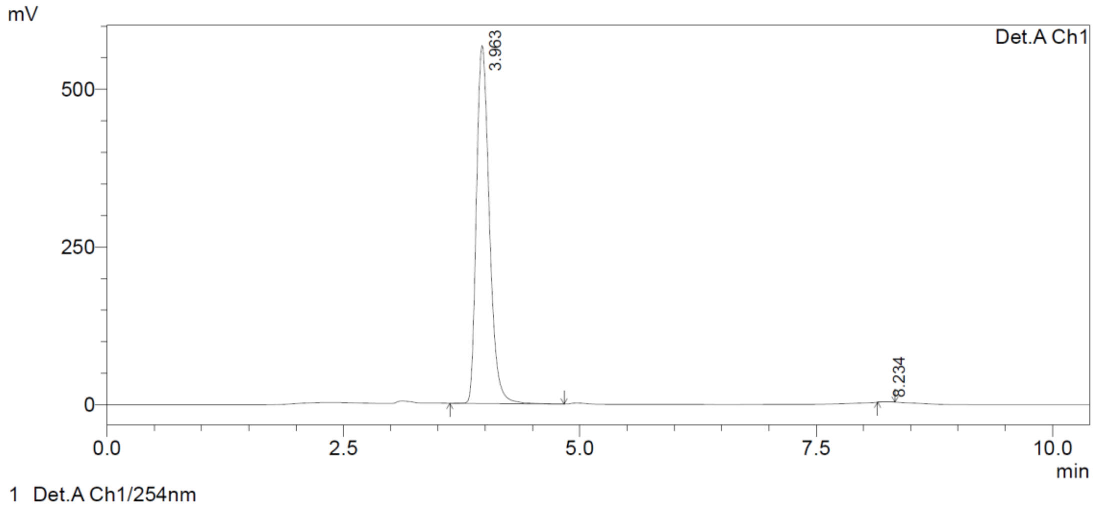

PeakTable

Detector A Ch1 254nm

Peak#	Ret. Time	Area	Height	Area %	Height %
1	3.963	5420227	567607	99.966	99.956
2	8.234	1826	249	0.034	0.044
Total		5422052	567856	100.000	100.000

AD-H, 1.0 mL/min, *n*-hexane/*i*-PrOH = 90 : 10, 254 nm, 99.97:0.03 er

Figure S11. HPLC spectrum of [(*S,S*)/(*R,R*)-diastereomers] after recrystallization

Spectra of (*S,R*)/(*R,S*)-diastereomers:

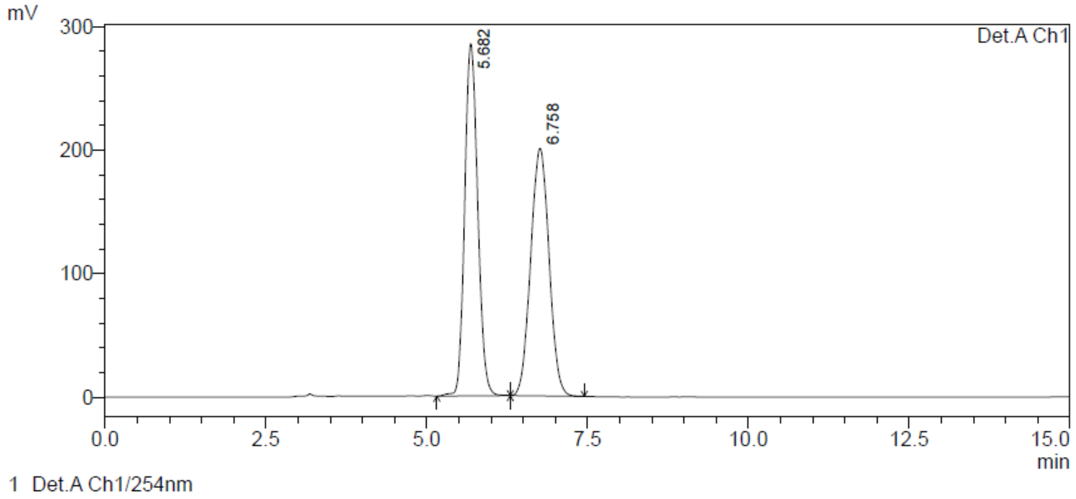

PeakTable

Detector A Ch1 254nm

Peak#	Ret. Time	Area	Height	Area %	Height %
1	5.682	4018953	285008	50.159	58.733
2	6.758	3993461	200255	49.841	41.267
Total		8012414	485263	100.000	100.000

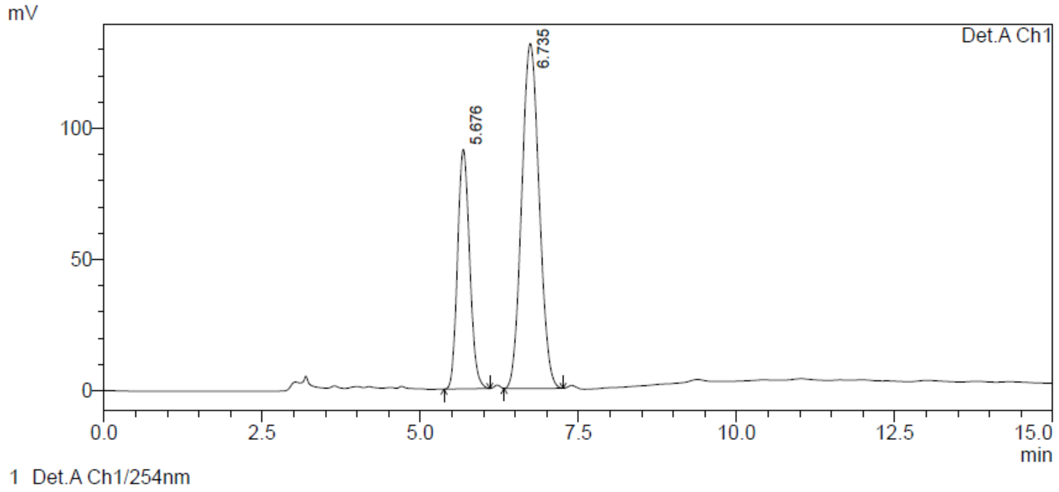

PeakTable

Detector A Ch1 254nm

Peak#	Ret. Time	Area	Height	Area %	Height %
1	5.676	1212593	91224	32.431	40.969
2	6.735	2526457	131440	67.569	59.031
Total		3739051	222664	100.000	100.000

AD-H, 1.0 mL/min, *n*-hexane/*i*-PrOH = 97 : 3, 254 nm, 68:32 er

Figure S12. HPLC spectrum of [(*S,R*)/(*R,S*)-diastereomers]

5. Reference

[1]. Choi, P. J.; Sutherland, H. S.; Tong, A. S. T.; Blaser, A.; Franzblau, S. G.; Cooper, C. B.; Lotlikar, M. U.; Upton, A. M.; Guillemont, J.; Motte, M.; Queguiner, L.; Andries, K.; Van den Broeck, W.; Denny, W. A.; Palmer, B. D. Synthesis and evaluation of analogues of the tuberculosis drug bedaquiline containing heterocyclic B-ring units. *Bioorg. Med. Chem. Lett.* **2017**, *27*, 5190–5196.

[2]. Ko, Y. K.; Im, C.; Do, J.; Park, Y. S. (+)-Sparteine-mediated substitution of *o*-benzyl-*N*-pivaloylaniline with ketones. *Eur. J. Org. Chem.* **2014**, 3460–3467.

[3]. Helmkamp, G. K.; Lucas, H. J. Stereochemistry of the reaction of 2,3-epoxybutane with alcohols. *J. Am. Chem. Soc.* **1952**, *74*, 951–954.

[4]. Perrona, Q.; Alexakis, A. Catalytic asymmetric bromine-lithium exchange: A new tool to build axial chirality. *Adv. Synth. Catal.* **2010**, *352*, 2611–2620.

[5]. Vincent Ming-Yau Leung, Matthew H. Gieuw, Zhihai Ke, Ying-Yeung Yeung. Intermolecular electrophilic bromoesterification and bromoetherification of unactivated cyclopropanes. *Adv. Synth. Catal.* **2020**, *362*, 2039–2044.
